# *JACMP* and the Problem of Success

**DOI:** 10.1120/jacmp.v16i4.5939

**Published:** 2015-07-08

Consider the following table that shows that the growth of the *JACMP* has averaged 20% year over year for the past 15 years:

**Table:** 

*Year*	*# Articles Peer‐Reviewed*	*# Articles Published*	*# Pages*	*% Growth Articles*	*% Growth Pages*
2000	unknown	19	164		
2001	unknown	29	232	53	41
2002	unknown	36	327	24	41
2003	unknown	45	385	25	18
2004	unknown	32	379	−29	−2
2005	unknown	44	492	38	30
2006	unknown	40	435	−9	−12
2007	83	48	592	20	36
2008	117	62	609	29	3
2009	164	80	817	29	34
2010	192	100	1098	25	34
2011	208	103	1229	3	12
2012	288	140	1484	36	21
2013	323	166	1805	19	22
2014	407	184	2199	11	22
Average annual growth over 15 yrs				19	21

The growth of 20% annually year over year has been very gratifying. It demonstrates that the *JACMP* is meeting a need and serving the interests of clinical medical physicists and their patients. With the sharp increase in complexity and technology in the 2000s, both imaging and oncology physicists needed a venue to publish solutions to time‐consuming and complex clinical physics problems. I believe the articles in the *JACMP* likely saved clinical physicists thousands of hours and allowed them to provide the benefits to patients many months earlier than would have been the case without the *JACMP*. Over these years, that has been the top priority — save time for clinical physicists and provide for our patients high‐quality treatments using these technologies as soon as possible.

Over the past 15 years the now‐defunct American College of Medical Physics (ACMP) and, beginning in 2012, the American Association of Physicists in Medicine (AAPM) have quietly maintained a most challenging business model. Articles published in the *JACMP* are still free to submit, free to publish, and free to access. However, there is a problem with this success. The growth in the number of pages and articles is not being matched by growth in revenue and income. The *JACMP* obviously costs money to create and publish. So, what are its resources?

One source is the revenue from banner ads. A second is a substantial subsidy from the AAPM. A third is the volunteer labor of the *JACMP* Editors over the years. But there are limits to all of these resources. There are twice the number of articles being published in 2015 as were published in 2011. The cost and number of volunteer hours consequently has increased proportionately. Indeed, this year the *JACMP* will publish approximately the same number of articles and pages as the *Medical Physics Journal* published in 2002.

So what is the solution? I believe any workable strategy will have multiple components and may be complex. An advertising page, such as found on the first page of printed *Medical Physics* articles, could generate additional revenue. Consolidating the two AAPM journals into a single platform and pricing the advertising according to the reach of both journals would also be helpful. Finally, the AAPM could institute some form of page or article charge for a *JACMP* publication. This latter solution is very common, in fact almost universal, for open‐access journals.

But are page/article charges the obvious solution for the *JACMP*? Here I do not want to run ahead of the AAPM Journals Business Management Committee (JsBMC), ably chaired by Sam Armato. The *JACMP* reach is world‐wide. Submissions source 30% from the US, 10% from Canada, and 60% from the rest of the world. Many physicists in developing nations might find that paying even a modest fee to publish an article is an insurmountable barrier. This is a problem the JsBMC will need to consider. I have confidence that the JsBMC will find the right solution for the AAPM and for the *JACMP*.

In the meantime, I want to thank the worldwide community that is the *JACMP*. We have over 6000 registered members and as many as 50,000 regular users, according to the number of IP addresses identified by Google analytics. The size and strength of the *JACMP* community, combined with the historical benefit to clinical physicists and their patients, give me confidence that some workable solution will be forthcoming.

Michael D. Mills, PhD

Editor‐in‐Chief

